# A novel surgical approach for fixation of a posterior chamber intraocular lens of Rayner 620 H with Gore-Tex suture

**DOI:** 10.1186/s12886-022-02759-3

**Published:** 2023-01-12

**Authors:** Tan Wang, Youxin Chen, Jun Lu, Ningning Li, Hanyi Min

**Affiliations:** 1 Department of Ophthalmology, Peking Union Medical College Hospital, Chinese Academy of Medical Sciences and Peking Union Medical College, No.1 Shuai Fu Yuan, Dongcheng District, 100730 Beijing, China; 2 Key Laboratory of Ocular Fundus Diseases, Chinese Academy of Medical Sciences and Peking Union Medical College, 100730 Beijing, China; 3 Department of Radiology, Affiliated Tumor Hospital of Zhengzhou University & Henan Cancer Hospital, 450008 Zhengzhou, Henan China; 4 Operating Room, Peking Union Medical College Hospital, Chinese Academy of Medical Sciences and Peking Union Medical College, 100730 Beijing, China

**Keywords:** Rayner IOL, Gore-Tex suture, Inadequate capsular support, Outcomes, Scleral-fixated intraocular lens

## Abstract

**Purpose:**

To report a novel surgical approach for the scleral fixation of the Rayner 620 H intraocular lens (IOL) with Gore-Tex suture and its outcomes at 6 months postoperatively.

**Methods:**

19 consecutive patients who underwent novel surgical approach for the scleral fixation of Rayner 620 H IOL with Gore-Tex suture at Peking Union Medical College Hospital between June 2020 and June 2021 were included. Data on best-corrected visual acuity (BCVA), spherical equivalent, total astigmatism/axis, short-term and long-term complications, and corresponding management with a follow-up of 6 months were collected.

**Results:**

Nineteen patients (11 men and 8 women) with a mean age of 62.7 ± 10.6 years were included. The median BCVA improved significantly from 0.90 ± 0.90 (Snellen 20/160) preoperatively to 0.20 ± 0.30 (Snellen 20/32) at postoperative 6 months follow-up (*P* < 0.001). The stratification of the accuracy of refractive outcomes was 53% of patients within ± 0.5 D and 84% of patients within ± 1.0 D of the refractive target. Corneal edema (*n* = 3, 16%) and increased intraocular pressure (IOP) (*n* = 4, 11%) were short-term complications. Long-term complications included increased IOP (*n* = 1, 5%), and macular edema (*n* = 1, 5%).

**Conclusion:**

The novel surgical approach for scleral fixation of the Rayner 620 H IOL with Gore-Tex suture is a reasonable option for patients who need secondary IOL placement without adequate capsular support.

## Introduction

In the absence of adequate capsular support, fixation of intraocular lens (IOL) is still challenging. Earlier surgical methods include placement of an anterior chamber IOL (ACIOL), iris-fixated IOL, or scleral fixation of posterior chamber IOL (PCIOL) with polypropylene suture [1, 2]. ACIOL placement may increase the risk of glaucoma, inflammation, and corneal decompensation [3]. For placement of an iris-fixated IOL, postoperative anterior chamber inflammation is considered a major concern due to the enclavation of iris tissue in the lens haptic [4], and the surgery is greatly restricted if the iris is injured by the trauma [5]. Scleral fixation of PCIOL with polypropylene suture has been associated with suture erosion with endophthalmitis, and late suture breakage leading to lens dislocation and exposure of the thread [6, 7].

Nowadays the traditional methods for fixating secondary lenses include scleral fixated with a Yamane or modified Yamane technique or with Gore-Tex sutures in a variety of ways [8–13]. It has been reported that scleral fixated with a Yamane technique has the advantages of being simple, a short surgery time, and firm IOL fixation, but it has the disadvantage that the surgical procedure is difficult and IOL tilt is relatively likely to occur. So various techniques have been reported to overcome these problems, and the modified technique can be selected according to the surgeon’s preference and environment [9, 14–16].

The Gore-Tex suture (W.L. Gore & Associates, Elkton, Maryland, USA), which is a nonabsorbable, polytetrafluoroethylene monofilament suture with greater tensile strength, minimal inflammatory response, and easy manipulation [17], has demonstrated favorable outcomes used for scleral fixation of IOLs [10, 11, 18]. For example, the one-year follow-up data of a novel surgical technique for the scleral fixation of a CZ70BDIOL (Alcon Laboratories, Inc, Fort Worth, TX) with Gore-Tex suture suggested that this technique is a reasonable surgical option for secondary IOL placement [10]. A prospective, interventional case series demonstrated that scleral fixation of IOLs with Gore-Tex suture was safe and well tolerated [11].

However, to the best of our knowledge, there has not yet been a study reporting a technique of scleral fixation with Gore-Tex suture for the widely distributed Rayner 620 H IOL. The purpose of this study was to propose an effective and simple approach for scleral fixation of the Rayner 620 H IOL with Gore-Tex suture and to report the outcomes of the surgical technique at a follow-up period of 6 months.

## Materials and methods

### Study design and population

We retrospectively studied 19 consecutive patients (11 men and 8 women, 19 eyes) who had undergone IOL (Rayner 620 H Spherical, Worthing, UK) fixation with a Gore-Tex suture between June 2020 and June 2021. Patients with 6 months of follow-up were included. All surgeries were performed by a single senior surgeon. For the IOL power calculations, preoperative axis length and corneal curvature were measured using optical biometry (IOLMaster 700; Carl Zeiss Meditec, Dublin, CA). The implanted IOL lens power allowed extrapolation of the surgeon’s target refraction in spherical equivalent based on Sanders-Retzlaff-Kraff theoretical (SRK/T). The target spherical equivalents of all the included subjects were set as 0°.

Preoperative data included age, sex, indications of surgery, other diseases of the eye, spherical equivalent, total astigmatism/axis, best-corrected visual acuity (BCVA), the position of the lens, and intraocular pressure (IOP). BCVA, spherical equivalent, total astigmatism/axis at 6 months after operation, the presence of any intraoperative or postoperative complications, and corresponding management were also recorded. The primary objective was to observe the changes in BCVA 6 months after surgery. The secondary objective was to observe the short-term and long-term complications after surgery. Complications occurring within 1 month after surgery were defined as short-term complications, whereas long-term complications were defined as complications that occurred more than 1 month after the procedure.

The procedure conformed to the tenets of the Declaration of Helsinki, and Institutional Review Board approval was obtained. Informed consent was obtained from all subjects and/or their legal guardian(s). All experimental protocols were approved by Ethics Committee of Drug Clinical Trials of Beijing Union Medical College Hospital.

### Procedure for the surgical technique

**Fig. 1:**
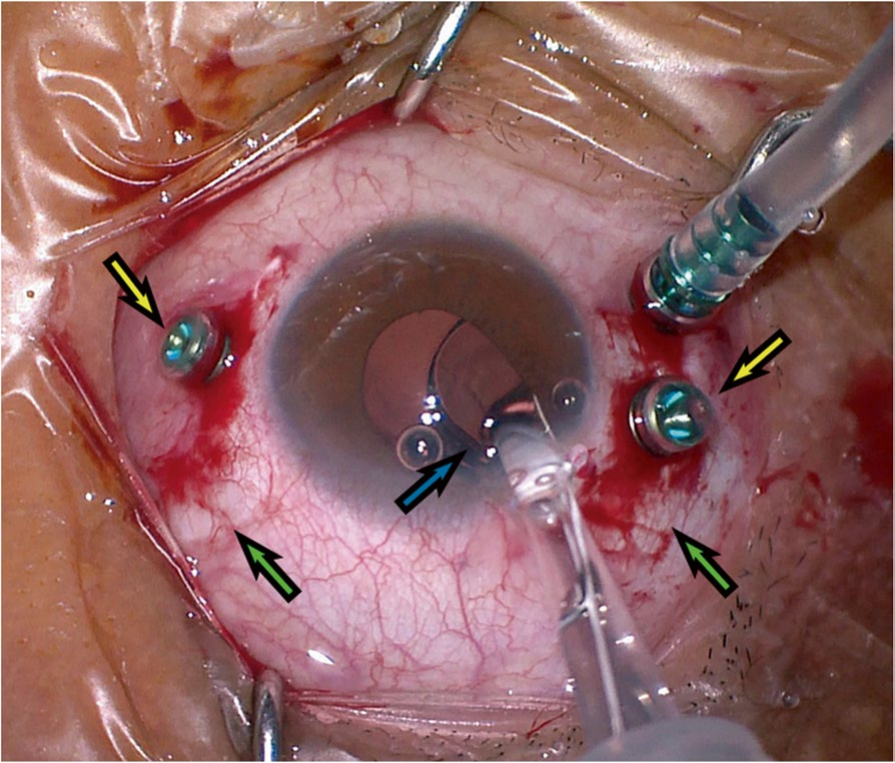
The position of conjunctival incision (green arrow) and sclerotomy (yellow arrow) separated by at least 1 o’clock. The IOL with the tying sutures was placed into the IOL injector (blue arrow) and introduced into the posterior chamber smoothly. IOL: intraocular lens

Using a superior approach, two 3-mm-long conjunctival radial incisions were created at the 2 o’clock and 10 o’clock positions, separately. On the left side, the conjunctival peritomy was made clockwise to 4 o’clock along the corneal limbus. On the right side, the peritomy was made to 8 o’clock counterclockwise. A standard infusion cannula was introduced approximately 3 mm posterior to the limbus (typically in the inferonasal quadrant, more than one o’clock away from the sclerotomies for suture fixation maneuvers). Persistent balanced salt solution (BSS) infusion was employed, and the IOP was maintained steadily at about 20 mm Hg. The other two cannulas were inserted at 3 and 9 o’clock vertically into the vitreous cavity and 2.5 mm away from the limbus (Fig. 1).

Generally, a toric lens marker was used to mark the corneal limbus at two points on the horizontal plane, 180° apart. The sclerotomy was made with a straight-entry, non-tunneled approach, and with the flat portion of the trocar blade parallel to the limbus. The pars plana vitrectomy (PPV) was performed through these entries. This technique is compatible with 23-, 25-, or 27-gauge instrumentation.

The anterior chamber was entered through the superior cornea using a 2.4 mm phaco keratome blade. The dislocated crystalline lens was removed completely by phacoemulsification and/or PPV depending on the individual. The vitreous body around the pupil was trimmed clearly by PPV. If the nucleus or cortex fell into the posterior cavity, a core PPV was performed, and the residual lens was removed completely. After PPV, the peripheral retina was examined in 360 degrees with scleral compressor.

If a patient had his/her own IOL dislocated in the vitreous cavity, a decision as to whether to preserve the dislocated IOL for suspending or change for another IOL was made. If the dislocated IOL was not suitable for suspending, it was taken from the vitreous cavity into the anterior chamber first and cut into several pieces by special scissors, then pulled out of the eye through the corneal incision completely. All residual capsule and cortex were removed by forceps and/or vitrectomized completely. A suitable Rayner 620 H IOL was prepared for implantation ahead of the operation. If the dislocated IOL was found to be suitable for suspension, it was taken into the anterior chamber first from the vitreous cavity after PPV. Then, the leading haptic was rotated out of the corneal incision, fastened with the Gore-Tex suture, and revolved back into the posterior chamber. The same process was repeated for the other haptic.

The needles of the 8 − 0 Gore-Tex suture (CV-8) were amputated, and the suture was cut into two halves. The haptics of the selected IOL were tied symmetrically in a 2-1-1 knot separately. Due to the hollow structure of two haptics and large diameter of the Rayner 620 H IOL, it was easier to determine the position of the knot bilaterally. The slippage of the knot, which was reported in three-piece IOLs of polymethyl methacrylate whose diameter of the haptics was equal or tapered [19], was not a concern. The IOL with the tying sutures can be placed into the IOL injector and introduced into the posterior chamber smoothly (Fig. 1). The sutures left in the injector were pulled out completely with forceps. In some conditions, the corneal incision can also be enlarged to implant the IOL by forceps directly.

**Fig. 2:**
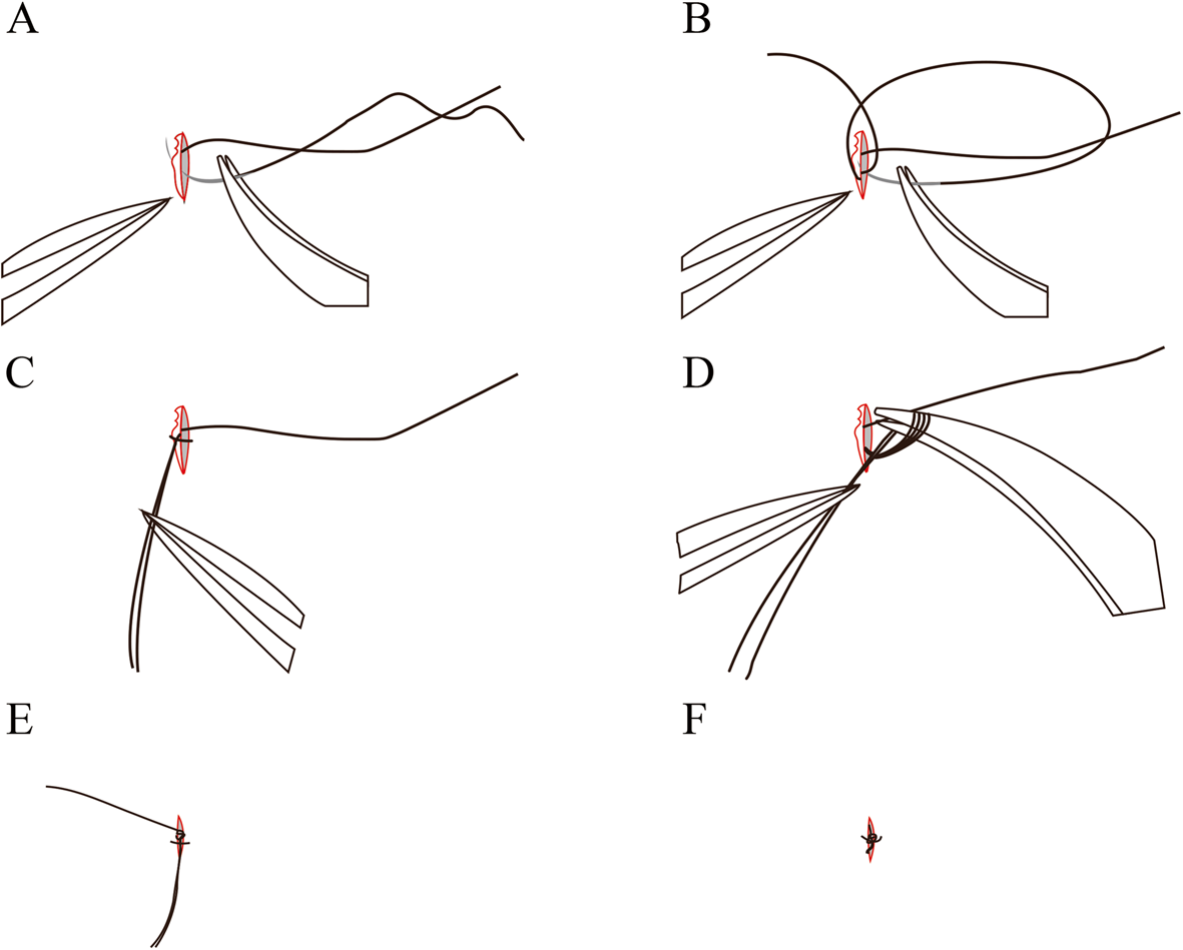
**A **The residual CV-8 suture left by the previous cut was taken and passed from the inside to the outside on the paranasal end of the incision. **B **The residual CV-8 suture was taken from the outside to the inside from the temporal end of the incision across the incision. **C** and **D** Then the ends of the head and tail of the residual CV-8 suture were put together and tied in a knot with the suspending suture that connected the haptic of the selected IOL. **E** and **F** The knot was buried mostly in the sclerotomy that previously housed the cannulas

Once introduced into the eye, the IOL was displaced into the posterior chamber. Under direct visualization, the two trailing nasal/temporal ends of the Gore-Tex suture were grasped and externalized through the respective sclerotomies using intraocular flat forceps. The two ends of the Gore-Tex suture were then pulled, and tension was balanced to ensure that the IOL optic was well centered. The trocars were then individually removed over the Gore-Tex suture. The suspending suture was used to tie a knot with the residual CV-8 suture in a simple interrupted mode. The knot was buried in the sclerotomy that previously housed the cannulas to minimize the chance of wound leakage (Fig. 2). If the end of the suture was not parallel to the surface of the sclera smoothly or even erected, a horizontal mattress of 8 − 0 Vicryl suture was used to flatten and fix on the surface of the sclera.

The viscoelastic material was then irrigated out of the anterior chamber, and the corneal incision was closed using the water-tight technique or a 10 − 0 Nylon suture. The overlying conjunctival peritomy was closed with an 8 − 0 Vicryl absorbable suture, ensuring that the Gore-Tex suture was completely covered.

**Fig. 3:**
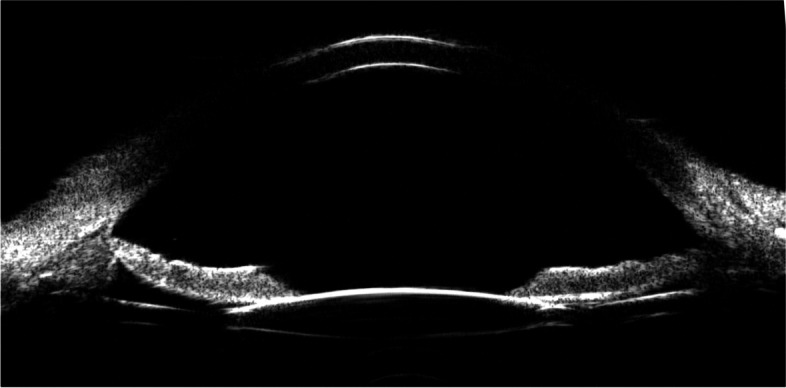
Ultrasound BioMicroscope image of the anterior segment after IOL implantation with Gore-Tex. The IOL was horizontal and centered. IOL: intraocular lens

Postoperatively, antibiotics and corticosteroids were administered routinely. The patients were followed at 1 week, 1 month, 3 months, and 6 months. Ultrasound BioMicroscope (UBM) imaging was performed at 1 month postoperatively to assess the centration and tilting of the IOL (Fig. 3).

### Statistical analysis

The International Standard visual acuities were converted to logarithmic minimum angle of resolution (logMAR) visual acuities [20]. Using SPSS 22.0 software (International Business Machines Corporation, New York), a normal distribution of data was verified using the Shapiro–Wilk test. The differences between preoperative and postoperative BCVA were analyzed using a paired two-tailed *t* test or Wilcoxon signed ranks test. A *P*-value less than 0.05 was considered statistically significant.

## Results

### General characteristics

Ten left eyes and nine right eyes of 19 patients (11 men and 8 women) with a mean age of 62.7 ± 10.6 years underwent the procedures during the 12-month study period. The included patients were followed for at least 6 months after surgery, with a mean follow-up of 236 ± 23 days. Individual patient data are summarized in Table 1.

**Table 1:** Data of the included patients

Patient	Age, years	Sex	Eye	Indication for surgery	Ocular comorbidity	Preoperative IOP, mm Hg	Preoperative spherical equivalent	POM 6 M spherical equivalent	Preoperative total astigmatism/axis	POM 6 M total astigmatism/axis	Preoperative BCVA	POM 6 M BCVA	Short-term complication (corresponding management)	Long-term complication (corresponding management)
1	68	F	OS	Subluxated/dislocated crystalline lens		20	-2.25D	0.50D	-2.00D/75	-1.50D/70	20/400	20/40		
2	51	M	OD	Subluxated/dislocated crystalline lens	Cataract; Transient ocular hypertension	18	-4.00D	-0.50D	-3.00D/140	-2.50D/160	20/20	20/25	Corneal edema (observation)	
3	53	M	OD	Dislocated IOL	Transient ocular hypertension	17	5.75D	1.25D	-1.00D/90	-1.00/95	20/100	20/25	Increased IOP (medication)	Increased IOP (medication)
4	42	M	OS	Dislocated IOL		22	2.50D	-0.50D	-1.00D/175	-1.50D/175	20/800	20/160		
5	58	M	OS	Subluxated/dislocated crystalline lens		16	3.50D	-1.00D	-2.00D/50	-1.00D/45	20/80	20/20		
6	56	F	OD	Subluxated/dislocated crystalline lens	Vogt-Koyanagi-Harada syndrome	18	-2.00D	0.00D	-1.50D/95	-1.00D/105	20/100	20/25		
7	79	F	OD	Subluxated/dislocated crystalline lens		10	-1.50D	-0.50D	-1.00D/40	-2.00D/40	20/20000	20/50		Macular edema (observation)
8	70	F	OD	Subluxated/dislocated crystalline lens		18	0.00D	0.00D	-0.50D/30	-0.50D/35	20/20000	20/50	Corneal edema (observation)	
9	56	F	OS	Dislocated IOL	Binocular uveitis; Behcet's disease	19	3.00D	-1.00D	-1.00D/80	-0.75D/80	20/125	20/30		
10	69	M	OD	Dislocated IOL	Cataract;	21	2.00D	-1.25D	-2.50D/110	-2.00D/105	20/800	20/114		
11	54	M	OS	Subluxated/dislocated crystalline lens	Cataract	17	-2.75D	-2.00D	-3.00D/10	-2.50D/15	20/125	20/32		
12	69	F	OD	Subluxated/dislocated crystalline lens		14	-4.00D	-0.50D	-2.50D/25	-2.50D/20	20/20000	20/25		
13	76	F	OS	Large intraoperative capsular break during cataract surgery		15	5.00D	0.50D	-5.00D/100	-4.50D/90	20/50	20/32		
14	66	M	OD	Subluxated/dislocated crystalline lens	Traumatic vitreous hemorrhage; Cataract	22	-6.00D	-1.00D	-0.50D/40	-0.50D/40	20/100	20/20	Corneal edema (observation)	
15	83	F	OS	Subluxated/dislocated crystalline lens		15	-2.50D	-1.00D	-2.50D/0	-2.50D/0	20/20000	20/100		
16	67	M	OS	Subluxated/dislocated crystalline lens	Marfan syndrome; Transient ocular hypertension	22	-0.50D	0.50D	-0.50D/60	-1.00D/65	20/160	20/32	Increased IOP (medication)	
17	55	M	OS	Subluxated/dislocated crystalline lens	Trauma	17	6.00D	1.00D	-2.00D/80	-1.75D/85	20/32	20/16	Corneal edema (observation)	
18	65	M	OD	Dislocated IOL	Cataract	12	-4.75D	0.50D	-3.50D/125	-3.00D/115	20/800	20/150		
19	55	M	OS	Aphakia (postoperative)	Transient ocular hypertension	23	8.50D	-1.00D	-1.00D/10	-1.50D/5	20/800	20/32	Increased IOP (medication); Corneal edema (observation)	

Abbreviations: *IOP* Intraocular pressure, *OD* Right eye, *OS* Left eye, *POM* postoperative month, *BCVA* Best-corrected visual acuity

The indications for surgery were subluxated/dislocated crystalline lens (12 patients, 63%), IOL dislocation (5 patients, 26%), aphakia secondary to complicated cataract extraction (1 patient, 5%), and large intraoperative capsular break during cataract surgery (1 patient, 5%).

### BCVA and refractive errors

The median preoperative logMAR BCVA of the 20 patients included in the analysis was 0.90 ± 0.90 (Snellen 20/160). The median logMAR BCVA with correctio at postoperative month 6 was 0.20 ± 0.30 (Snellen 20/32); this change was statistically significant (*P* < 0.001). The average preoperative spherical equivalent and postoperative spherical equivalent were − 1.32D ± 4.77D and − 0.34D ± 0.89D, respectively. The average preoperative total astigmatism and postoperative total astigmatism were − 1.90D ± 1.20D and − 1.76D ± 1.00D, respectively. The target spherical equivalents of all the included subjects were set as 0° before the operation. The stratification of the accuracy of refractive outcomes was 53% of patients within ± 0.5 D and 84% of patients within ± 1.0 D of the refractive target.

### Short-term complications

The most common short-term complication was corneal edema (5 patients, 26%), which resolved within the first few weeks after surgery and did not require additional intervention. Increased IOP (16%) was found in three patients, and temporary medications were applied to control IOP, so it returned to normal within a short period of time.

### Long-term complications

**Fig. 4:**
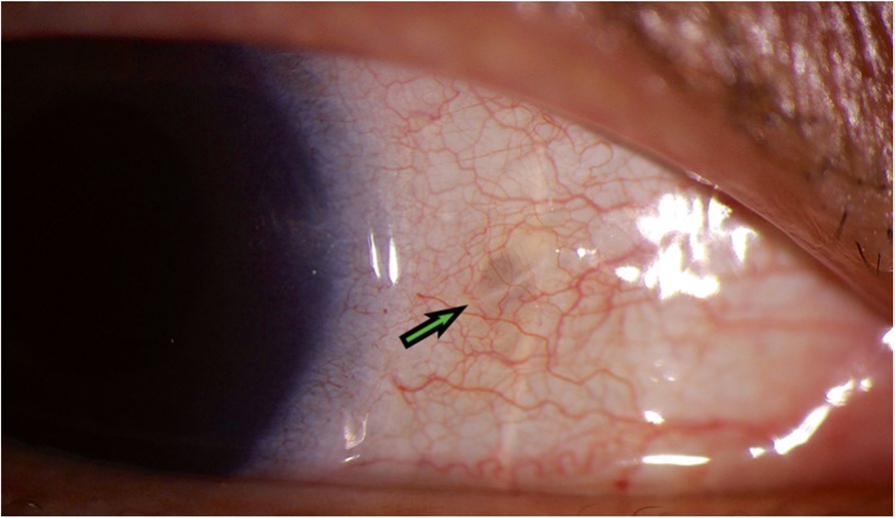
Representative case 6 months after operation with Gore-Tex. The bilateral Gore-Tex suture lied smoothly on the surface of the sclera and could be observed under the conjunctiva (green arrow)

Increased IOP was found in one patient (5%), and macular edema was also found in one patient (5%). After a short period of observation, these patients returned to normal. At 6 months postoperatively, the bilateral Gore-Tex suture of all patients lied smoothly on the surface of the sclera, no exposed sutures were found (Fig. 4).

## Discussion

The changes in visual acuity and the short-term and long-term complications after surgery suggest that scleral fixation with Gore-Tex suture for Rayner 620 H IOL is safe and reliable. The characteristics of this surgical method are as follows: ① Gore-Tex suture has great advantages over a polypropylene suture surgically; ② The outer knot is buried into the sclerotomy tunnel and no scleral flap is created; ③ The Rayner 620 H IOL is foldable and can be implanted with a bolus injection device after tying, so most of the corneal incision used to implant the IOL does not need to be sutured; ④ The two haptics with hollow structure which allowing the fixation with the 2-1-1 knot symmetrically and the large diameter of the Rayner 620 H IOL are able to avoid displacement or oblique of IOL; ⑤ the distance of conjunctival incision and sclerotomy is more than 1 o’clock (Fig. 1).

The tensile strength of the Gore-Tex suture makes it durable during surgery and allows for more permanent fixation of the IOL [21, 22]. There have been reports of polypropylene suture erosion and degradation (13.8%—breakage time of about 40.8 months) [7]. Polypropylene suture breakage has been attributed to both suture cutting by haptic-positioning holes and degradation of the polypropylene material itself [23–25]. In addition, the polypropylene suture is hard, thin, and easy to expose out of the conjunctiva. In contrast, Gore-Tex is an expanded monofilament polytetrafluoroethylene suture, and it has been demonstrated to remain intact in vivo for decades [13]. It is also white and can clearly be seen and handled during operation; the 8 − 0 Gore-Tex suture can prevent the cutting effect on the sclera and the hepatics of the IOL more effectively because it has a larger diameter (0.040–0.049 mm) than the more commonly used 10 − 0 polypropylene suture (0.020–0.029 mm) or 9 − 0 polypropylene suture (0.030–0.039 mm).

However, Gore-Tex suture has been reported that if the knot ends of Gore-Tex sure protrude through the conjunctiva, it may cause granuloma formation or possible infection and require IOL removal [26]. A previous study reported that burying the suture in the scleral groove can reduce the rate of suture erosion and endophthalmitis related to the erosion [27]. In the current study, the scleral knot can be buried completely in the sclerotomy, and the residual suture can be flat on the surface of the sclera. Unlike the methods of scleral fixation of IOL with polypropylene suture, our technique does not require making a scleral flap or tunnel. Moreover, we made the scleral and conjunctival incisions at least 1 o’clock away from each other; in such cases, severe inflammation occurred rarely, and the tail of the threads of almost all cases was well under the conjunctiva. Thus, displacement of the conjunctival incision and a sclerotomy at least 1 o’clock apart is strongly recommended in these kinds of surgeries. Such a displacement of the two incisions could not only make the Gore-Tex suture covered well by the conjunctiva, but it would also greatly reduce postoperative inflammation compared to the way that the two incisions were at the same position. In addition, this method is easy to operate and may need a short operation time. However, in patients with relatively thin conjunctiva or when the conjunctiva is patchy, a scleral flap or tunnel is recommended.

The Rayner 620 H IOL is widely used, and the foldable IOL can be inserted into a bolus injection device. When using the bolus injection device to inject IOL, a limbal-corneal incision is short and water-tight without suture. In addition, the two haptics are cubic columns. After fixation, one side can fit the inner surface of the eyeball to close the puncture port, maintain IOP stability, and avoid any cutting effects on the eyeball tissue.

Decentration and tilting of the IOL, which would cause the gaze point to shift sideways, radial astigmatism, and changes of spherical power are important concerns in IOL implantation [28]. Intraoperative and postoperative IOP fluctuation also affect the position of the IOL. Nowadays the traditional methods for fixating secondary lenses include scleral fixated with a Yamane or modified Yamane technique or with Gore-Tex sutures in a variety of ways [8–13]. The Yamane technique has the disadvantage that the IOL tilt is relatively likely to occur, so various techniques have been reported to overcome these problems, and the modified technique can be selected according to the surgeon’s preference and environment [9, 14–16]. In addition, several methods of scleral fixation with Gore-Tex sutures in a variety of ways, such as the cow-hitch knot, in-and-out technique, and four-point fixation, may ensure the centration of a variety of IOLs [10, 12, 28, 29]. The Rayner 620 H IOL has been widely distributed in the world. The design of the haptics is suitable for suture. The structure of the haptics is hollow so that the knot is easily, accurately, and symmetrically fixed in two tails, and no slippage of the knot can take place. Once tying the bilateral thread simultaneously to fix them against the eyewall, the IOL will be kept centrally and horizontally well. Generally, it is unnecessary to modify the bilateral tension of the two threads unlike in other methods. In our series, the IOL was centered well, and no tilting was found by general examination and UBM imaging postoperatively. Figure 2 shows centration and horizontal position of the IOL under UBM.

There are studies reporting that sulcus fixation leads to a better centration than pars plana fixation because the diameter of the IOL exceeds the mean diameter of the ciliary sulcus in eyes with an average length (11.1 mm) [28, 30]. The diameter of Rayner 620 H IOL is 12.5 mm and exceeds the sulcus diameter, so the decentration caused by the position of the sclerotomy 2.5 mm away from the limbus is minimal. Even if it is skewed, the large refractive center (6.5 mm) will ensure that visual acuity is not affected. However, if sclerotomy is fixed in the par plana plane or 3 mm longer away from the limbus, the tension between the bilateral haptics should be modified carefully to balance the lens. To some degree, a greater diameter of the IOL is associated with easier maintenance of its centration.

Compared to the previously reported technique of scleral-fixated posterior chamber intraocular lenses of the Rayner 620 H IOL [31], there are several differences in the current study. First, the difference in the type of sutures used in the current surgical approach was different. As described in the manuscript, the Gore-Tex suture has great advantages over a polypropylene suture surgically. Second, we made the scleral and conjunctival incisions at least 1 o’clock away from each other. Such an approach was to make the Gore-Tex suture covered well by the conjunctiva and reduce postoperative inflammation caused by the corrosive effect on the conjunctiva. Third, the Rayner 620 H IOL was generally implanted with a bolus injection device after tying to reduce damage to the corneal incision and lighten the postoperative changes in corneal refractive power.

There were some limitations to our study. The sample size of this study was small, and the follow-up time was relatively short. Another limitation is the retrospective and single-center study design.

In summary, fixation of the Rayner 620 H IOL with Gore-Tex suture is a novel, effective, and simple approach for patients requiring IOL fixation with inadequate capsular support.

## Data Availability

The datasets used and/or analysed during the current study available from the corresponding author on reasonable request.

## References

[1] Vasavada AR, Nihalani BR (2006). Pediatric cataract surgery. Curr Opin Ophthalmol.

[2] Holt DG, Young J, Stagg B, Ambati BK (2012). Anterior chamber intraocular lens, sutured posterior chamber intraocular lens, or glued intraocular lens: where do we stand?. Curr Opin Ophthalmol.

[3] Dick HB, Augustin AJ (2001). Lens implant selection with absence of capsular support. Curr Opin Ophthalmol.

[4] Messina M, Elalfy M, Fares U (2016). Creeping posterior synechiae following hyperopic iris-fixated phakic implants. Int Ophthalmol.

[5] Kim EJ, Brunin GM, Al-Mohtaseb ZN (2016). Lens Placement in the absence of capsular support: scleral-fixated Versus Iris-fixated IOL Versus ACIOL. Int Ophthalmol Clin.

[6] Vote BJ, Tranos P, Bunce C, Charteris DG, Da Cruz L (2006). Long-term outcome of combined pars plana vitrectomy and scleral fixated sutured posterior chamber intraocular lens implantation. Am J Ophthalmol.

[7] Wasiluk E, Krasnicki P, Dmuchowska DA, Proniewska-Skretek E, Mariak Z (2019). The implantation of the scleral-fixated posterior chamber intraocular lens with 9/0 polypropylene sutures - long-term visual outcomes and complications. Adv Med Sci.

[8] Yamane S, Ito A (2021). Flanged fixation: Yamane technique and its application. Curr Opin Ophthalmol.

[9] Randerson EL, Bogaard JD, Koenig LR (2020). Clinical outcomes and Lens constant optimization of the Zeiss CT Lucia 602 Lens using a modified Yamane technique. Clin Ophthalmol.

[10] Bonnell AC, Mantopoulos D, Fine HF (2020). One-year outcomes of a Novel Surgical Approach for fixation of a posterior Chamber intraocular Lens using Gore-Tex suture. Retina.

[11] Bhojwani D, Vasavada AR, Vasavada V (2020). Intraoperative performance and long-term postoperative outcomes after scleral fixation of IOLs with polytetrafluoroethylene suture. J Cataract Refract Surg.

[12] Fan K, Patel N, Laura D, et al. Refractive outcomes of four point scleral fixation of Akreos A060 intraocular Lens using Gore-tex suture. Investigative Ophthalmology & Visual Science; 2019. p. 60. 10.2147/OPTH.S282094 PMC7762441 33376297

[13] Khan MA, Samara WA, Gerstenblith AT (2018). COMBINED PARS PLANA VITRECTOMY AND SCLERAL FIXATION OF AN INTRAOCULAR LENS USING GORE-TEX SUTURE: One-Year Outcomes. Retina..

[14] Tang Y, Gao Y, Chu Y, Liu Y, Han Q (2022). Modified Yamane technique with a 26-gauge needle: single corneal incision and simplified haptic insertion. J Cataract Refract Surg.

[15] Yavuzer K, Evcimen Y, Tang Y (2019). Sutureless transconjunctival intrascleral intraocular lens fixation: the modified Yamane technique modified Yamane technique with a 26-gauge needle: single corneal incision and simplified haptic insertion. Arq Bras Oftalmol.

[16] Bhatia K, Manaktala R, Sachdev M (2021). MYX technique: a modified adaptation of Yamane and extraocular needle-guided haptic insertion techniques for scleral-fixated intraocular lens implantation. Indian J Ophthalmol.

[17] Khan MA, Gupta OP, Smith RG (2016). Scleral fixation of intraocular lenses using Gore-Tex suture: clinical outcomes and safety profile. Br J Ophthalmol.

[18] Huang CW, Tsai CY, Lai TT (2021). Short-term outcomes of a modified technique for small-incision scleral-fixated intraocular lens implantation using Gore-Tex sutures. Graefes Arch Clin Exp Ophthalmol.

[19] Hayashi K, Hayashi H, Nakao F, Hayashi F (1998). Comparison of decentration and tilt between one piece and three piece polymethyl methacrylate intraocular lenses. Br J Ophthalmol.

[20] Holladay JT (1997). Proper method for calculating average visual acuity. J Refract Surg.

[21] Bizzarri F, Tudisco A, Ricci M, Rose D, Frati G (2010). Different ways to repair the mitral valve with artificial chordae: a systematic review. J Cardiothorac Surg.

[22] Shimamoto T, Komiya T, Sakaguchi G (2011). Half-leaflet suspension with a thin Gore-Tex suture for aortic leaflet prolapse. Ann Thorac Surg.

[23] Price MO, Price FW (2005). Late dislocation of scleral-sutured posterior chamber intraocular lenses. J Cataract Refract Surg.

[24] Parekh P, Green WR, Stark WJ, Akpek EK (2007). Subluxation of suture-fixated posterior chamber intraocular lenses a clinicopathologic study. Ophthalmology.

[25] Drews RC (1983). Polypropylene in the human eye. J Am Intraocul Implant Soc.

[26] Bhojwani D, Vasavada AR, Vasavada V (2020). Intraoperative performance and long-term postoperative outcomes after scleral fixation of IOLs with polytetrafluoroethylene suture. J Cataract Refract Surg.

[27] Almashad GY, Abdelrahman AM, Khattab HA, Samir A (2010). Four-point scleral fixation of posterior chamber intraocular lenses without scleral flaps. Br J Ophthalmol.

[28] Choi JY, Han YK (2018). In-and-out technique for intraocular lens scleral fixation. Clin Ophthalmol.

[29] Patel NA, Shah P, Yannuzzi NA (2018). Clinical outcomes of 4-point scleral fixated 1-piece hydrophobic acrylic equiconvex intraocular lens using polytetrafluoroethylene suture. Clin Ophthalmol.

[30] Ohmi S, Uenoyama K, Apple DJ (1992). [Implantation of IOLs with different diameters]. Nippon Ganka Gakkai zasshi.

[31] Kaczmarek IA, Prost ME, Wasyluk JT (2018). Comparison of Retropupillary Iris-claw intraocular Lens Implantation and Transscleral suture fixation of an intraocular Lens for Aphakic Eyes. J Clin of Diagn Res..

